# Diffuse EGFR staining is associated with reduced overall survival in locally advanced oesophageal squamous cell cancer

**DOI:** 10.1038/sj.bjc.6602625

**Published:** 2005-06-28

**Authors:** L Gibault, J-P Metges, V Conan-Charlet, P Lozac'h, M Robaszkiewicz, C Bessaguet, N Lagarde, A Volant

**Affiliations:** 1 Department of Anatomical Pathology, Hepato-Gastroenterology, La Cavale Blanche Hospital, Boulevard Tanguy Prigent, 29609 Brest Cedex, France; 2 Institute of Oncology, Morvan Hospital, 5 Avenue Foch, 29609 Brest Cedex, France; 3 Department of General Surgery, Hepato-Gastroenterology, La Cavale Blanche Hospital, Boulevard Tanguy Prigent, 29609 Brest Cedex, France; 4 Department of Hepato-Gastroenterology, Hepato-Gastroenterology, La Cavale Blanche Hospital, Boulevard Tanguy Prigent, 29609 Brest Cedex, France; 5 Department of Biostatistics and Cancer Register Unit, Hepato-Gastroenterology, La Cavale Blanche Hospital, Boulevard Tanguy Prigent, 29609 Brest Cedex, France

**Keywords:** oesophageal cancer, EGFR, VEGF, immunohistochemistry, HER-2, ckit

## Abstract

Squamous cell carcinoma of the oesophagus (SCCO) is still a pathology of bad prognosis. Specific therapies are now developed against epidermal growth factor receptor (EGFR), human epidermal growth factor receptor 2, c-kit receptor (CD117), vascular endothelial growth factor (VEGF) and p53 protein. This study was aimed at assessing their expression in a large series of SCCO, as well as their potential therapeutic interest in this pathology. Immunohistochemical expression of these factors was assessed retrospectively in 107 cases of SCCO with primary surgery, as well as their relationships to recurrence, metastasis and overall survival on a long-term follow-up. Human epidermal growth factor receptor 2 and CD117 were expressed in less than 3% of the cases. Epidermal growth factor receptor and p53 were overexpressed in 68.2 and 66.4% of the cases, and VEGF in 38.3%. Epidermal growth factor receptor overexpression was significantly related to vascular invasion (*P*=0.023). Its diffuse positivity was significantly related in multivariate analysis to higher local recurrence (*P*=0.006) and lower overall survival (*P*=0.003), in a subgroup of patients of poor outcome who had received postoperative adjuvant treatment. These results highlight the great potential prognostic and therapeutic interest of evaluating EGFR diffuse positivity in locally advanced SCCO.

Squamous cell carcinoma of the oesophagus (SCCO) still has a bad prognosis. Improving the survival of SCCO patients is nowadays a challenge for practitioners confronted with this pathology. Several options in treatment are available and remain debated among oncologists, despite the use of standardised surgical procedures and the development of promising chemotherapy agents such as Taxans and Irinotecan (Ilson *et al*, 1999; Metges *et al*, 2001).

In addition to these drugs, novel therapies are nowadays developed, specifically directed against growth factor receptors (Mendelsohn and Baselga, 2000; Grünwald and Hidalgo, 2003) and angiogenesis factors (Falm, 2004). These factors are noticeably interesting targets for therapy, as they seem to be frequently involved in carcinogenesis pathways. This led us to wonder about their implication in oesophageal carcinogenesis and potential use as therapeutic targets in this pathology.

We therefore assessed their immunohistochemical expression in surgical specimens from SCCO patients within a geographical area (Brittany, France), where the incidence of this cancer is noticeably high (Benhamiche *et al*, 1999), as well as their relationship to prognosis. Three of the factors studied (epidermal growth factor receptor (EGFR), human epidermal growth factor receptor 2 (HER-2) and CD117) are tyrosine kinase receptors, defined by an extracellular ligand-binding specific site, a transmembrane domain and an intracellular domain with tyrosine-kinase activity. Epidermal growth factor receptor and HER-2 belong to the c-erb receptor family (Downward *et al*, 1984; Coussens *et al*, 1985), and are implicated in cell proliferation, differentiation and migration in physiological processes. In cancerogenesis, their overexpression leads to abnormal cell proliferation (Ullrich and Schlessinger, 1990; Klapper *et al*, 2000; Olayioye *et al*, 2000). Epidermal growth factor receptor can be overexpressed through diverse and mainly post-transcriptional mechanisms. It can be targeted by specific monoclonal antibodies, for example, *IMC-C225* (Mendelsohn and Baselga, 2000), which is directed against the extracellular domain of the receptor. Phase-II and phase-III trials with this molecule have been performed in patients with progressive disease, in addition to chemotherapy, in refractory colorectal (Saltz *et al*, 2004), or head and neck (Trigo *et al*, 2004) cancers. As for HER-2, it is mostly overexpressed through gene amplification and can be targeted by a monoclonal antibody, *trastuzumab,* which has proved to be quite promising in metastatic breast cancer patients (Slamon *et al*, 2001).

CD117 (also known as c-kit) is a type-III tyrosine kinase receptor with five extracellular, immunoglobulin-like domains. This protein is strongly involved in myelopoiesis, melanogenesis and spermatogenesis (Yarden *et al*, 1987; Chabot *et al*, 1988). In some cancers such as gastro intestinal stromal tumours (GISTs), point mutations lead to abnormal expression and constitutive activity of the receptor. CD117 is targeted there by *imatimib mesylate* (Heinrich *et al*, 2002).

Our investigations also focused upon the expression of a growth factor, vascular endothelial growth factor (VEGF), involved in physiological and pathological angiogenesis (Connolly *et al*, 1989; Ferrara, 2002); it is now targeted by *bevacizumab*, a humanised monoclonal antibody (Presta *et al*, 1997). Phase III randomised trials have been undertaken; the first results in metastatic colorectal cancer tend to prove a partial efficiency (Hurwitz *et al*, 2004).

We eventually studied the expression of the p53 protein, known to be mutated in numerous cancers. The mutated p53 protein loses its ability to regulate cell proliferation (Crawford *et al*, 1981). New genetic therapies are presently developed in order to restore the physiological function of p53 (Edelman *et al*, 2003).

## PATIENTS AND METHODS

### Patients included in the study

Our study was conducted retrospectively. We selected patients who had undergone primary surgery for a SCCO between 1987 and 1997. All of them had been operated on by the same surgeon (PL), trained in oesophageal surgery. A Lewis-Santy procedure with two-field lymphadenectomy was used in all cases. Patients who had received preoperative treatment, those with metastasis at the time of diagnosis and the ones with other histological types than a SCC were excluded. We also excluded patients who had died from postoperative complications and patients with macroscopically or microscopically incomplete surgical resection. This to a total of 107 patients finally included in the study.

This population was termed ‘SP’ for ‘Study Population’. Survival analyses were performed in this population. SP patients were also divided into two groups ‘pot+’ (*n*=48) and ‘pot−’ (*n*=59), depending on whether a postoperative combined radio- and chemo therapy had been administered or not. Decisions concerning postoperative treatment were made in a multidisciplinary weekly meeting that included gastroenterologists, oncologists, surgeons and pathologists. ‘Pot+’ patients consisted of two groups: those who presented high risk of recurrence and/or metastasis after surgery, and those receiving radio- and chemotherapy as treatment for local recurrence and/or metastasis occurrence. Chemotherapy regimens consisted in an association of 5-fluorouracil (800 mg m^−2^ day^−1^, days 1–5) and cisplatin (15 mg m^−2^ day^−1^, days 1–5), whereas the radiotherapy schedule was a split course regimen with 3 gy day^−1^, 5 days a week, on weeks 1 and 3. On the opposite, ‘pot−’ patients did not receive any postoperative adjuvant treatment. This group was mainly composed of patients considered as presenting low risk of local recurrence or metastasis (pT1, T2 and N0 stages), but it also included some advanced patients with an altered general condition that forbade any adjuvant treatment.

### Follow-up

Follow-up ended on December 31, 2002. It consisted of medical history and medical examination with haematological and biochemical tests, chest radiography, abdominal ultrasonography, endoscopies with biopsies of surgical anastomosis and CT scan. Exams were performed every 6 months for the first 2 years, every year until the fifth year, and then every 2 years, most of the time for 10 years. For the few patients who were lost to follow-up in both the Surgical and Oncology Departments, we collected data from their gastroenterologist, family doctor or from the Cancer Register Unit.

### Immunohistochemical staining methods

For each patient, we retrieved Bouin-fixed and paraffin-embedded archival tissue samples. Tissue sections (4-*μ*m-thick) were deparaffinised and rehydrated in water. For antigen retrieval, samples were incubated 40 min at 100°C in a citrate buffer at pH 6. Endogenous peroxidase activity was blocked for 15 min with 0.3% hydrogen peroxide. Then, sections were rinsed with phosphate-buffered saline (PBS) and incubated with selected primary antibodies as follows: (i) a polyclonal antibody from rabbit (Santa Cruz Biotech®, 1/100 dilution and 45-min incubation) for VEGF; (ii) polyclonal antibodies against HER-2 (Dako®, 1/1000 dilution, 30-min incubation) and against CD117 (Dako®, 1/400 dilution, 20-min incubation); (iii) mouse monoclonal antibodies against human EGFR (2-18C9 clone, Dako®, from Dako EGFR pharmDx™ kit K1494 and K1492, 30-min incubation) and human p53 protein (D0-7 clone, Dako®, 1/50 dilution, 10-min incubation). After incubation, all samples were rinsed with PBS. Those stained with p53, VEGF, HER-2 and CD117 were incubated with a biotinilised secondary antibody (‘LINK’ from Dako® for p53, HER-2 and CD117; Amersham® for VEGF); the streptavidin–biotin method was applied for signal detection. Epidermal growth factor receptor primary antibody was labelled with the diaminobenzidine (DAB) system after incubation with the secondary antibody. All slides were then rinsed and counterstained with haematoxylin.

### Staining evaluation

Immunohistochemical staining was assessed by two pathologists (LG and AV) who worked independently of each other at first, then reviewed the slides together. For p53 and CD117, a significant staining of more than 10% of tumour cells was considered as positive. As done for breast cancer, HER2 staining was evaluated in a semiquantitative way with a cutoff of 10% ‘3+’ cells (Penault-Llorca *et al*, 2002). Tumour cells were considered to be ‘EGFR positive’ when their staining was more marked than that of the adjacent normal epithelium, in agreement with previous interpretations of EGFR in SCCO (Yano *et al*, 1991; Itakura *et al*, 1994). Whenever they had been found positive, we evaluated the percentage of cells displaying the same intensity of staining. Staining pattern was qualified as ‘diffuse’ when intensity was alike in more than 95% of cancer nests, and termed as ‘mosaic’ in the case of heterogeneous intensity of staining. Vascular endothelial growth factor staining was considered as positive when more than 30% of tumour cells were stained more intensely than normal smooth muscle cells, according to a reference interpretation (Inoue *et al*, 1997).

### Statistical analysis

A *χ*^2^-test was carried out to analyse the relationships between the expression of the studied factors and other clinical parameters. Survival, local recurrence and metastasis were studied in the study population ‘SP’, and in ‘pot+’ and ‘pot−’ groups. Patients who had died from causes other than their oesophageal cancer were censored for survival analysis. Survival curves were assessed with the Kaplan–Meier analysis for censored data, and compared with the log rank test in univariate analysis. The confidence level for significance was set at *P*<0.05. A Cox regression model for multivariate analysis was applied in an ascending step-by-step method for factors within a significant level of 0.20 in univariate analysis. Statistical analyses were conducted with the SPSS® software (12.0 version).

## RESULTS

### Characteristics of the studied population

‘SP’ consisted of 94 men and 13 women (sex ratio 7/1). Patients ranged from 42 to 79 years old. In accordance with the World Health Organisation Classification standards (Gabbert *et al*, 2000), 44 patients (41.1%) presented a well-differentiated tumour, 51 a moderately differentiated one (47.7%) and 12 a poorly differentiated one (11.2%). The depth of invasion, according to TNM classification (Gabbert *et al*, 2000), was variable, with a majority of pT3 stages (*n*=61, 57.0%). Vascular invasion and lymph node involvement were, respectively, observed in 35 (32.7%) and 41 cases (38.3%).

Concerning ‘pot+’ and ‘pot−’ groups, ‘pot+’ patients displayed, as suggested by the therapeutic indications, a more locally advanced disease (higher proportion of pT3 and N1 stages as compared with the ‘pot−’ group). Outcome variables such as local recurrence or metastasis occurrence were significantly increased in this group (*P*=0.028 and 0.016, respectively).

Detailed characteristics of SP, ‘pot+’ and ‘pot−’ groups are summarised in Tables 1 and 2.

### Immunohistochemical staining

**Figure 1:**
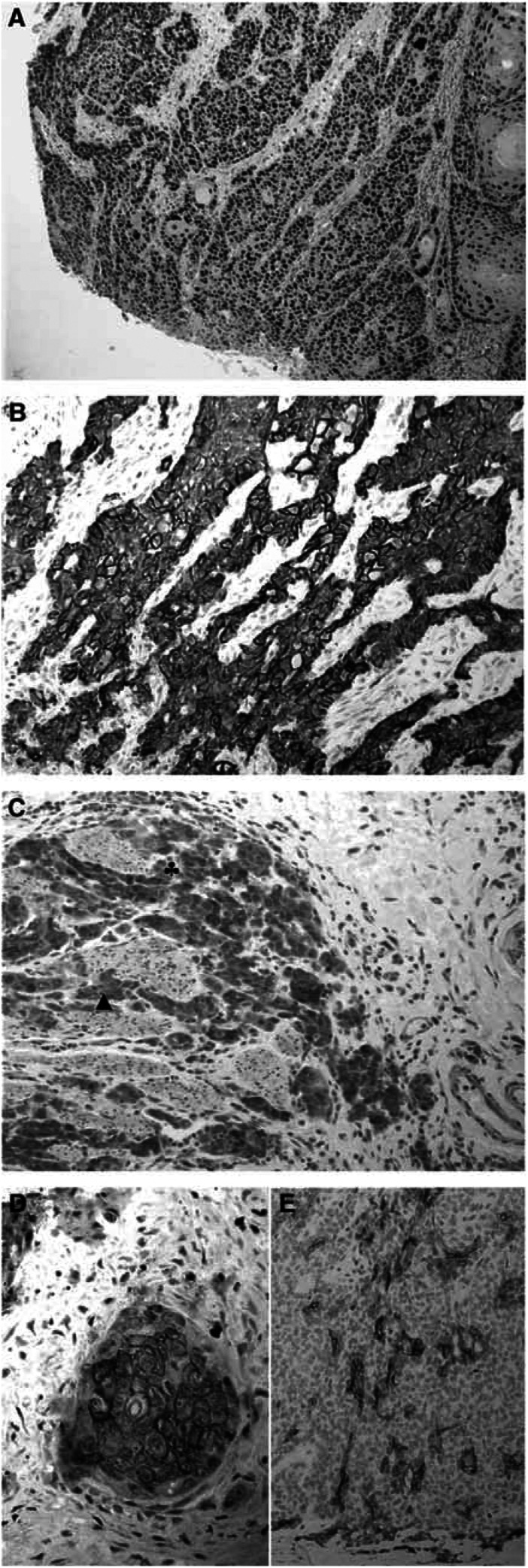
Immunohistochemical expression of carcinogenesis factors in SCCO: (**A**) p53 intense nuclear staining, HESx100, (**B**) EGFR intense membranous staining, HES × 200, (**C**) VEGF expression: cytoplasmic staining, more intense in cancer cells (♣) than in smooth muscle cells (▴), HES × 200; (**D**) HER-2 complete and strong membranous staining, scored ‘3+’, HES × 400; (**E**) CD117 focal cytoplasmic staining, HES × 200.

In all, 73 patients (68.2%) displayed a moderate to intense expression of EGFR in cancer cells. Immunohistochemical staining was restricted to cancer cell membranes. A total of 55 tumours (51.4%) were stained in a ‘mosaic’ pattern and 18 (16.8%) in a ‘diffuse’ one. Nuclear positivity for p53 was found in 71 cases (66.4%); VEGF was diffusely expressed in 41 cases (38.3%). On the contrary, HER-2 and CD117 were expressed in only three cases (2.8%). Human epidermal growth factor receptor 2 staining intensity was heterogeneous. For CD117, the staining was cytoplasmic, often faint and focal; all tumours were surrounded with normal, strongly positive mastocytes (Figure 1).

### Relationships with clinical parameters

*χ*^2^-tests were conducted in the SP (Table 3). They evidenced a statistically significant relationship between EGFR expression and vascular invasion (*P*=0.023, data not shown); most of the cases with vascular invasion displaying EGFR staining, regardless of the staining pattern. In addition to this, VEGF expression was related to tumour differentiation (*P*=0.012), this factor being rarely expressed in well-differentiated tumours. Human epidermal growth factor receptor 2 and CD117 were too rarely expressed to establish conclusive and statistically significant relationships. A *χ*^2^-test on EGFR, p53 and VEGF expressions found no significant relationship between them.

### Prognosis studies

At the end of follow-up, 84 patients (79.5%) had died, 22 others (20.6%) were alive and one (0.9%) had been lost to follow-up. Among the deceased patients, six died from associated throat cancer, one from lung cancer, nine others from heart failure (*n*=2), haematemesis (*n*=1), intestinal infarction (*n*=1), inhalation (*n*=1), altered condition (*n*=1), delirium tremens crisis (*n*=1), suicide (*n*=1) and car accident (*n*=1). These 16 patients (15.9%) died from causes that were not directly linked to their oesophageal cancer or its treatment. They were therefore censored for survival analysis. Prognosis was not studied for HER-2 or CD117+ patients: the very low number of involved subjects invalidated statistical comparisons with the log-rank test. Local recurrence and metastasis studies were not studied in the ‘pot−’ group for the same reasons.

### Survival study

In the SP group, the median survival time was 37 months [18–56]. In univariate analysis, survival was significantly related to pT stage (*P*=0.001), vascular (*P*=0.001) and lymph node (*P*<0.001) invasion. In multivariate analysis, only pT stage and lymph node invasion were linked to prognosis (*P*=0.027 and 0.007) (Table 4). Expressions of the factors studied were not significantly linked to survival in SP.

The ‘pot−’ group demonstrated a median survival time of 82 months [20–144]. Only pT2-T3 stages (*P*=0.014), vascular (*P*=0.004) or lymph node invasion (*P*<0.001) were related to a reduced survival in univariate analysis. In multivariate analysis lymph node invasion was associated with a poorer survival (*P*=0.002) (Table 4).

In the ‘pot+’ group, median survival time was 21 months [11–31]. Patients with advanced pT stage had a significant poorer survival in multivariate analysis (*P*=0.038). For EGFR-expressing patients, this difference was not found significant in univariate analysis (*P*=0.186). However, patients displaying a ‘diffuse’ expression of EGFR had a significantly reduced survival, both in univariate (*P*=0.006) and multivariate analysis (*P*=0.003) (Table 4).

### Local recurrence study

In SP, median recurrence-free survival (RFS) was 108 months. A significant relationship was established between a reduced RFS and parietal invasion (*P*=0.075), vascular invasion (*P*=0.028) and lymph node involvement (*P*<0.001) in univariate analysis. Patients who did not express p53 or who expressed EGFR tended to have a decreased RFS, but the difference was not significant (*P*=0.073 and 0.079, respectively). In multivariate analysis (Table 5), only lymph node involvement remained significant (*P*=0.005).

In the ‘pot+’ group, the median RFS was 56 months [23–89]. No clinical or pathological parameter was related to prognosis. Patients with either EGFR expression or diffuse EGFR expression had a shorter RFS than the other ones (*P*=0.125 and 0.096, respectively).

In multivariate analysis (Table 5), only EGFR diffuse expression was significantly related to a shorter RFS, compared to negative staining for EGFR (*P*=0.006).

### Metastasis occurrence study

In SP, median metastasis-free survival (MFS) was 46 months [16–75]. Significant relationships were established between a shorter MFS and parietal invasion (*P*=0.050), vascular invasion (*P*<0.001) and lymph node involvement (*P*<0.001). In the case of EGFR expression or diffuse EGFR expression, this relationship was present, but failed to reach statistical significance (*P*=0.075 and 0.109). Only lymph node involvement was significant in multivariate analysis (*P*<0.001) (Table 5).

In the ‘pot+’ group, median MFS was 17 months [13–20]. Patients with pT2 or pT3 stages had a reduced MFS (*P*=0.038), as well as patients with lymph node involvement (*P*<0.001), and patients expressing EGFR in a diffuse pattern (*P*=0.016). Patients expressing EGFR, regardless of the staining pattern, had a shorter MFS with no statistically significant difference (*P*=0.061). In multivariate analysis, only lymph node involvement was significantly related to prognosis (*P*=0.004) (Table 5).

## DISCUSSION

### Originality of our work

Immunohistochemistry is nowadays a simple, reproducible way to assess the expression of oncogenic factors in paraffin-embedded samples from cancer tissues. It is therefore more and more used customarily to study the expression of new potential therapeutic targets and to determine which patients are the most liable to answer to these specific therapies.

To our knowledge, our series is among the few studies focused on the *simultaneous* assessment, in SCCO, of five of the most interesting molecular targets in oncology and on their relationship to prognosis. Our series is also quite interesting because of its homogeneity in therapeutic procedures (primary Lewis-Santy surgery, similar postoperative treatment in subgroups of patients), and for its long follow-up (from 5 to 15 years), with only one patient lost to follow-up.

Indications of postoperative adjuvant treatment in SCCO still remain controversial. We believe that, among patients with advanced disease, it can improve disease-free and overall survival. Several studies support this hypothesis: Bedard and Coll (2001) demonstrated, in a retrospective study, a significant better survival in lymph-node positive patients having benefited from postoperative chemoradiation therapy, as compared to surgery alone. Ando and Coll multicenter randomized controlled trial (Ando *et al*, 2003) also evidenced an improved disease-free survival in SCCO patients having benefited from adjuvant chemotherapy.

In our study, ‘pot+’ patients, who received postoperative chemoradiation, had nonetheless a significantly worse prognosis. However, this worse prognosis was mainly due to the indications of treatment: as shown in Table 2, these patients had significantly more often vascular or lymph node invasion and most of them were treated for local recurrence or metastasis occurrence. Therefore, this group constituted an interesting collection of individuals of advanced stages, where the usual prognosis markers such as lymph node involvement and parietal invasion were not sufficient to predict prognosis.

### Epidermal growth factor receptor in SCCO

Among the factors studied, the EGFR appears to be outstanding in SCCO. According to literature data, EGFR overexpression in this pathology ranges from 45.6 to 72.1% (Itakura *et al*, 1994; Shimada *et al*, 1999); our results are consistent with these findings. Most of the studies found a significant relationship to metastasis occurrence (Shimada *et al*, 1999), lymph node involvement and survival (Yano *et al*, 1991). By using EGF-binding assays, Ozawa and Coll (1989) and Mukaida and Coll (1991) drew the same conclusions, and so did Kitagawa and Coll (1996) from slot-blot hybridation experiments.

In our work, EGFR expression, evaluated in accordance with previous standards, was not found significant in prognosis studies, although the patients expressing EGFR tended to have a poorer prognosis. However, evaluation of EGFR ‘diffuse’ expression has revealed particularly interesting, even in a long-term follow-up. This ‘diffuse’ *vs* ‘mosaic’ scoring system has rarely been conducted in SCCO. Yano and Coll (1991) and Itakura and Coll (1994) already used it in SCCO; more recently, Wilkinson and Coll (2004) also assessed it in adenocarcinoma of the oesophagus. In our series, it was found to be significantly related in multivariate analysis to a worse prognosis in an assortment of patients of advanced stages. It seemed to be also associated to a reduced recurrence-free survival, as well as to a reduced metastasis-free survival in univariate analysis.

As EGFR is also a potential therapeutic target, evaluation of EGFR overexpression is paramount in cancer strategy. Among the different molecules against EGFR that have been developed, IMC-C225, directed against the extracellular domain of the receptor, inhibits EGFR ligand-binding and autocrine activation and should be promising in patients overexpressing EGFR. However, in refractory colorectal cancer, EGFR immunohistochemical overexpression, with a rate of 1% tumoral positive cells for a positive score, did not predict response to this therapy (Saltz *et al*, 2004). Such a scoring system may not then be entirely relevant, and the therapeutic implications due to technical procedures (especially in the assessment of EGFR status before treatment) clearly emphasise the need for consensus publications. According to our results, further studies of diffuse and mosaic EGFR expression may allow the identification of different subgroups of patients liable to be good candidates for EGFR therapies, especially among patients with advanced disease. Above all, prospective works assessing EGFR diffuse expression and its relationship to clinical response to EGFR-targeted therapies are definitely needed in oesophageal cancer.

### P53 protein in SCCO

Our study evidenced a high expression of p53 in SCCO. This has been well established in previous studies conducted with nearly the same immunohistochemical procedures, together with a lack of correlation to prognosis, which is consistent with our findings (Sarbia *et al*, 1994; Hardwick *et al*, 1997; Shimada *et al*, 1999; Wang *et al*, 1999; Ahn *et al*, 2002; Rosa *et al*, 2003). P53 mutations are peculiarly frequent in high-SCCO incidence areas like Brittany (Audrezet *et al*, 1993), where we performed our study; some geographic specificity might also be implicated. Further studies are needed to explore the role of p53 in SCCO in these regions, and the potential impact of p53 activity-restoring therapies.

### Vascular endothelial growth factor in SCCO

Vascular endothelial growth factor was overexpressed in about 40% of our cases. In SCCO, its immunohistochemical expression is variable among studies and ranges from 23.9 (Ogata *et al*, 2003) to 68.9% (Shimada *et al*, 1999). Its relationship to prognosis also remains unclear. Indeed, according to most of the available studies, VEGF expression significantly predicts a poorer survival (Inoue *et al*, 1997; Uchida *et al*, 1998; Koide *et al*, 1999, 2001; Kato *et al*, 2002). On the contrary, some other recent reports (Ahn *et al*, 2002; Rosa *et al*, 2003) are consistent with our results, evidencing a lack of relationship between VEGF expression and clinical outcome in SCCO. Underneath this may prevail a difficulty in immunohistochemical interpretation of VEGF staining. Inoue *et al*'s (1997) reference method for staining interpretation seems to be the most consistent with VEGF overexpression physiopathology: VEGF is defined as positive only whenever it is more intensely expressed in tumour cells than in normal smooth muscle cells. Only few studies (Ahn *et al*, 2002; Rosa *et al*, 2003) used this method, which permits comparison with our findings. Further studies about homogeneous groups of patients, with reference technique and staining interpretation, should cast a precise light on VEGF implication in oesophageal carcinogenesis and its potential interest as a therapeutic target. Interestingly, VEGF expression was significantly related to survival in a previous study we conducted in a homogeneous population of gastric adenocarcinomas (Fondevila *et al*, 2004), where we applied Inoue's interpretation method.

### Human epidermal growth factor receptor 2 and CD117 expression

The immunohistochemical evaluation of HER-2 and CD117 is now quite well standardised. Our follow-up evidenced a very seldom expression of both HER-2 and CD117 in SCCO. The use of external or internal positive controls (positive breast cancer for HER-2, positive mastocytes in the cancer stromal reaction for CD117) during immunohistochemical procedures rules out the hypothesis of false-negative reactions.

For HER-2, these results are consistent with other studies (Shiga and Coll, 1993), but we lack of comparative data since, to our knowledge, studies concerning HER-2 or CD117 expressions are very scarce. According to our findings, these factors nevertheless appear to be of poor interest as potential therapeutic targets in SCCO.

## CONCLUSIONS

Understanding of biological processes implicated in carcinogenesis is a new asset in the treatment of cancer. In Brest University Hospital, a multidisciplinary team made up of oncologists, gastroenterologists, surgeons and pathologists has grown for several years very concerned about oesophageal cancer. Our wide series of SCCO patients granted us enough material to conduct a retrospective study of 107 patients, with particularly long follow-up (from 5 to 15 years), and only one patient lost to follow-up. This was the ideal medium to assess simultaneously the expression of five major molecular targets in cancerology, as well as their relationship to prognosis.

We could therefore demonstrate that EGFR diffuse expression, in a subgroup of patients with more advanced disease, was significantly related to a lower overall survival and higher local recurrence in multivariate analysis. On the contrary, p53 protein and VEGF were overexpressed in our series, but not related to prognosis. As for HER-2 and c-kit receptor, they were very rarely expressed.

Epidermal growth factor receptor would therefore be a good potential target for specific therapies, especially those directed against the extracellular domain of the receptor, such as IMC-C225. There is still controversy about the relevance of EGFR scoring in selecting patients for EGFR-directed therapies. However, evaluation of EGFR ‘diffuse’ *vs* ‘heterogeneous’ expression has seldom been performed, and has never been related to clinical response to EGFR-targeted therapies in SCCO. Further prospective works and clinical trials concerning EGFR-directed therapies, assessing EGFR diffuse positivity, are needed. If they confirmed our results, they would bring new hopes in the clinical outcome of SCCO.

## Figures and Tables

**Table 1:** Characteristics of the study population

**Clinical parameters and outcome variables**	**Study population**
*Age*	
<55 years old	32 (29.9%)
55–65 years old	42 (39.3%)
>65 years old	33 (30.8%)
	
*Gender*	
Male	94 (87.9%)
Female	13 (12.1%)
	
*Tumour differentiation*	
Well differentiated	44 (41.1%)
Moderately differentiated	51 (47.7%)
Poorly differentiated	12 (11.2%)
	
*pT*	
pT1	34 (31.8%)
pT2	12 (11.2%)
pT3	61 (57.0%)
	
*pN*	
N−	66 (61.7%)
N+	41 (38.3%)
	
*Vascular invasion*	
No	72 (67.3%)
Yes	35 (32.7%)
	
*Local recurrence*	
No	71 (66.4%)
Yes	36 (33.6%)
	
*Metastasis occurrence*	
No	56 (52.3%)
Yes	51 (47.7%)
	
*Postoperative treatment*	
No (pot− group)	59 (55.1%)
Yes (pot+ group)	48 (44.9%)
Total	107 (100%)

**Table 2:** Characteristics of the ‘pot+’ and ‘pot−’ populations

	**‘Pot−’ group**		**‘Pot+’ group**		
**Clinical parameters and outcome variables**	**Nb**	**% (95% CI)**	**Nb**	**% (95% CI)**	***P***
*Age*					
<55 years old	18	30.5% [18.8–43.3]	14	29.2% [16.3–42.0]	0.894
55–65 years old	22	37.3% [24.9–44.6]	20	41.6% [27.7–55.6]	
>65 years old	19	32.2% [20.3–44.1]	14	29.2% [16.3–42.0]	
					
*Gender*					
Male	54	91.5% [84.4–98.6]	40	83.3% [72.8–93.9]	0.197
Female	05	08.5% [02.8–18.7]	08	16.7% [07.5–30.2]	
					
*Tumour differentiation*					
Well differentiated	26	44.1% [31.4–56.7]	18	37.5% [23.8–51.2]	0.777
Moderately differentiated	27	45.7% [33.1–58.5]	24	50.0% [35.9–64.1]	
Poorly differentiated	06	10.2% [03.8–20.8]	06	12.5% [04.7–25.3]	
					
*pT*					
pT1	22	37.3% [24.9–49.6]	12	25.0% [12.8–37.3]	0.150
pT2	10	17.0% [08.4–29.0]	05	10.4% [03.5–22.7]	
pT3	27	45.7% [33.1–58.5]	31	64.6% [51.1–78.1]	
					
*pN*					
N0	41	69.5% [57.7–81.2]	25	52.1% [38.0–66.2]	0.065
N1	18	30.5% [18.8–42.3]	23	47.9% [33.8–62.0]	
					
*Vascular invasion*					
No	45	76.3% [65.4–87.1]	27	56.2% [42.2–70.3]	0.028
Yes	14	23.7% [12.9–34.6]	21	43.8% [29.7–57.8]	
					
*Local recurrence*					
No	45	76.3% [65.4–87.1]	26	54.2% [40.1–68.3]	0.016
Yes	14	23.7% [12.9–34.6]	22	45.8% [31.7–59.9]	
					
*Metastasis*					
No	45	76.3% [65.4–87.1]	11	22.9% [11.0–34.8]	<0.001
Yes	14	23.7% [12.9–34.6]	37	77.1% [65.2–89.0]	
Total	59	100%	48	100%	

Nb=number of patients, 95% CI=95% confidence interval, *P*=significance of the difference between ‘pot−’ and ‘pot+’ groups.

**Table 3:** Expression of the studied factors according to clinical parameters

	**EGFR expression**				**P53 expression**			**VEGF expression**		
**Clinical parameters and outcome variables**	**Neg**	**M pos**	**D pos**	***P***	**Neg**	**Pos**	***P***	**Neg**	**Pos**	***P***
*Age*										
< 55 yo	10	18	04	*0.299*	09	23	*0.731*	15	17	*0.091*
55–65 yo	10	25	07		15	27		27	15	
> 65 yo	14	12	07		12	21		24	09	
										
*Gender*										
Male	33	46	15	*0.087*	31	63	*0.937*	59	35	*0.752*
Female	01	09	03		05	08		07	06	
										
*Diff*										
WD	12	21	11	*0.124*	18	26	*0.126*	34	10	*0.012*
MPD	22	34	07		18	45		32	31	
										
*pT*										
pT1	14	16	04	*0.312*	09	25	*0.284*	23	11	*0.386*
pT2-T3	20	39	14		27	46		43	30	
										
*pN*										
N0	24	33	09	*0.196*	19	47	*0.177*	42	24	*0.598*
N+	10	22	09		17	24		24	17	
										
*Vasc. Inv.*										
No	28	32	12	*0.061*	23	49	*0.593*	48	24	*0.128*
Yes	06	23	06		13	22		18	17	
										
*Local recurrence*										
No	25	35	11	*0.552*	20	51	*0.092*	44	27	*0.931*
Yes	09	20	07		16	20		22	14	
										
*Metastasis occurrence*										
No	21	28	07	*0.278*	21	35	*0.376*	34	22	*0.829*
Yes	13	27	11		15	36		32	19	
										
*EGFR status*										
Negative		12	22	*0.800*	21	13	*0.268*			
M pos		17	38		31	24				
D pos		07	11		14	04				
										
*P53 positivity*										
Negative			19	17	*0.177*					
Positive			47	24						
Total	34 31.8%	55 51.4%°	18 16.8%		36 33.6%	71 66.4%		66 61.7	41 38.3	

yo=years old, Diff=differenciation, WD=well differentiated, MPD=moderately to poorly differentiated, Vasc. Inv.=vascular invasion, M pos=mosaic positivity, D pos=diffuse positivity, neg=negative, pos=positive.

**Table 4:** Independent prognosis factors in multivariate analysis for overall survival in SP, ‘pot−’ and ‘pot+’ group

	***P***	**Odd ratio**	**95% CI**
*SP*			
Parietal invasion	0.027	1.96	1.1–3.6
Lymph node involvement	0.007	2.02	1.2–3.3
			
*Pot*−			
Lymph node involvement	0.002	3.64	1.6–8.1
			
*Pot*+			
EGFR ‘mosaic’ positivity	0.535	1.27	0.6–2.7
EGFR ‘diffuse’ positivity	0.003	4.52	1.7–12.2
pT2-T3 invasion	0.038	2.19	1.0–4.6

95% CI=95% confidence interval.

**Table 5:** Independent prognosis factors in multivariate analysis for local recurrence and metastasis occurrence, in SP and ‘pot+’ group

**Groups**	**Local recurrence**	***P***	**Odd ratio**	**95% CI**
SP	Lymph node involvement	0.005	2.68	1.3–5.3
Pot+	EGFR ‘mosaic’ positivity	0.161	2.45	0.70–8.59
	EGFR ‘diffuse’ positivity	0.006	9.62	1.90–48.76
	**Metastasis occurrence**			
SP	Lymph node involvement	<0.001	4.08	2.3–7.2
Pot+	Lymph node involvement	0.001	3.08	1.6–6.1

95% CI=95% confidence interval.
